# Algae and Cyanobacteria Fatty Acids and Bioactive Metabolites: Natural Antifungal Alternative Against *Fusarium* sp.

**DOI:** 10.3390/microorganisms13020439

**Published:** 2025-02-17

**Authors:** Miguel E. López-Arellanes, Lizbeth Denisse López-Pacheco, Joel H. Elizondo-Luevano, Georgia María González-Meza

**Affiliations:** 1 School of Engineering and Sciences, Tecnológico de Monterrey, Monterrey 64700, Nuevo León, Mexico; a00838140@tec.mx (M.E.L.-A.); lizdenissepacheco@gmail.com (L.D.L.-P.); 2 Institute of Advanced Materials for Sustainable Manufacturing, Tecnológico de Monterrey, Monterrey 64700, Nuevo León, Mexico; 3 Faculty of Agronomy, Universidad Autónoma de Nuevo León, San Nicolás de los Garza 66455, Nuevo León, Mexico; joel.elizondolv@uanl.edu.mx

**Keywords:** cyanobacteria, microalgae, *Fusarium*, fatty acids, antifungal activity, bioactive molecules, phenolic acids

## Abstract

Fungal diseases caused by *Fusarium* spp. significantly threaten food security and sustainable agriculture. One of the traditional strategies for eradicating *Fusarium* spp. incidents is the use of chemical and synthetic fungicides. The excessive use of these products generates environmental damage and has negative effects on crop yield. It puts plants in stressful conditions, kills the natural soil microbiome, and makes phytopathogenic fungi resistant. Finally, it also causes health problems in farmers. This drives the search for and selection of natural alternatives, such as bio-fungicides. Among natural products, algae and cyanobacteria are promising sources of antifungal bio-compounds. These organisms can synthesize different bioactive molecules, such as fatty acids, phenolic acids, and some volatile organic compounds with antifungal activity, which can damage the fungal cell membrane that surrounds the hyphae and spores, either by solubilization or by making them porous and disrupted. Research in this area is still developing, but significant progress has been made in the identification of the compounds with potential for controlling this important pathogen. Therefore, this review focuses on the knowledge about the mechanisms of action of the fatty acids from macroalgae, microalgae, and cyanobacteria as principal biomolecules with antifungal activity, as well as on the benefits and challenges of applying these natural metabolites against *Fusarium* spp. to achieve sustainable agriculture.

## 1. Introduction

One of the objectives of the United Nations 2030 Sustainable Development Agenda is to preserve the life of terrestrial ecosystems and eradicate hunger; both objectives are related to new practices for more sustainable agriculture and more responsible food production. Phytopathogens inflict substantial damage, causing up to 30% of the diseases that affect more than 150 food crops, including cereals, vegetables, and fruits [1]. The global threat of fungal pathogens in crops results in a dramatic loss of yield, along with a significant impact on food quality, leading to multiple cascading effects and economic losses. The most prevalent phytopathogens that affect crops before and after harvest are *Ustilago* spp., *Sclerotinia* spp., *Pestalotiopsis* spp., *Alternaria* spp., *Rhizoctonia* sp., *Colletotrichum* sp., *Botrytis* sp., *Phytophthora* sp., and *Fusarium* spp. [2].

*Fusarium* is a genus comprising more than 100 species of filamentous fungi; some are considered the most severe crops phytopathogens. *Fusarium* has a wide host range and is difficult to control due to its ability to develop resistance, survive in the soil for long periods, persist within agricultural residues following harvest, and cause health damage with its toxins [3]. The main problems caused by *Fusarium* infection in plants are vascular wilting, root and stem rot, fruit rot, and mycotoxin contamination. Mycotoxins, which are secondary metabolites produced by *Fusarium*, such as trichothecenes, zearalenone, enniatins, deoxynivalenol, and fumonisins, pose a significant threat to health. Ingestion of these metabolites can harm both animals and humans through exposure to contaminated food [4,5].

Certain species of *Fusarium* have the highest incidence and impact on crops. They include *Fusarium oxysporum*, which causes vascular wilt in a wide range of crops [6], *Fusarium graminearum*, which primarily leads to discoloration of cereal ears and contamination with mycotoxins [7], and *Fusarium solani*, which is known for causing root and stem rot in crops such as potatoes, tomatoes, and beans [8]. Other species, such as *Fusarium culmorum*, *Fusarium equiseti*, *Fusarium gallinaceum*, and *Fusarium chlamydosporum*, are also commonly found in cereal crops, which cause considerable damage. Therefore, it is important to highlight that the incidence and impact of different *Fusarium* spp. vary depending on the type of crop, environmental conditions, and agricultural practices [9,10].

Implementing integrated control strategies against *Fusarium* spp. can help reduce the incidence and impact of this phytopathogen on agricultural crops. Traditionally, crop rotation, the use of resistant crop varieties, and effective soil management practices are employed to prevent or manage *Fusarium* spp. [11]. However, the use of synthetic fungicide products remains a quick and effective strategy. Some of the most used chemical fungicides against *Fusarium* spp. in crops include triazoles, strobilurins, carbendazim, mancozeb, benomyl, tebuconazole, and propiconazole [12]. Unfortunately, the excessive and indiscriminate use of these chemicals can lead to environmental and health problems, such as the development of resistance to fungicides in certain strains, increased carcinogenic effects in humans, and residual toxicity in the environment [13]. Concerns about the adverse effects of fungicides have prompted regulations that require compliance with specific production standards in the sector. This has motivated scientific researchers to utilize biotechnology in the search for promising, environmentally friendly natural alternatives.

The research studies point to the development of sustainable biological product technologies for control in agriculture. These products focus on biofungicides, biological bactericides, biostimulants, and other products derived from plant molecules and microorganisms. Biological control of phytopathogens involves identifying and utilizing various biological control agents with potential fungicidal activity. These agents include microorganisms such as bacteria and fungi, as well as plant-derived compounds from algae, lichens, and higher plants. Their antifungal effects operate through different mechanisms [14]. Furthermore, microorganisms act as antagonists to fungi by naturally competing for essential nutrients and space for colonization, thereby limiting the development of the involved phytopathogen [15]. Additionally, a study identified *Bacillus* strains that produced antifungal compounds, such as lipopeptides, as well as enzymes that inhibited the growth of *F. equiseti* [14]. Micoparasitism is another biofungicide mechanism in which microorganisms parasitize fungal hyphae to absorb their nutrients. In vitro tests showed that *Trichoderma asperellum* secretes cell wall-degrading enzymes, like chitinases and cellulases associated with mycoparasitic activity, against *F. oxysporum* in stevia plants, which reduces *Fusarium* wilt incidence by 80% [16].

An advantage of using microorganism biofungicides is that some biocontrol agents can promote plant defenses in addition to limiting the growth of phytopathogens. *Bacillus subtilis* has demonstrated an 85.3% inhibition ratio against *Fusarium* spp. in potatoes while also providing biostimulant properties [17]. A study published by Zhao et al. (2021) showed that it inhibited the mycelial growth of *F. oxysporum* by 81.3%. Furthermore, it was observed that *Penicillium bilaiae* significantly promoted the growth of sesame [18].

Some of the most effective control agents against *Fusarium* spp, with potential under greenhouse or field conditions, belong to the genera *Trichoderma*, *Bacillus*, *Pseudomonas*, and *Streptomyces* [19,20]. However, biomass production of these microorganisms in bioreactors and synthetic culture media can significantly increase costs, which represents a challenge for bioprocess scale-up.

An alternative is the antifungal activity of plant extracts or specific or secondary plant metabolites, which has also been studied. In vitro studies of these compounds are the first phase of bioprospecting, which is followed by the development of new fungicide products. These compounds are found in herbs and plants [21]. Some natural compounds that have shown efficacy against *Fusarium* in vitro include cinnamaldehyde and phenylpropanoids, which are present in cinnamon essential oil and have demonstrated 92.98% disease suppression [22]; the monoterpenes, which are present in oregano oil [23]; sulfur compounds, which are present in garlic extracts; and the oxygenated monoterpenes, which are present in turmeric oil [24]. However, cultivating terrestrial plants to extract antifungal compounds competes with the demand for food and, in turn, requires arable land and water, making it an unsustainable future strategy with environmental impact.

In this context, macroalgae, microalgae, and cyanobacteria, which produce bioactive metabolites, have shown promise as effective agents for the biological control of plant pathogens and the improvement of soil fertility but without the harmful effects or environmental impact of other methods. Algae and cyanobacteria are photosynthetic organisms capable of synthesizing bioactive compounds, such as peptides, fatty acids, polysaccharides, phenolic compounds, terpenes, alkaloids, and sulfur compounds, thus demonstrating antimicrobial and antifungal properties [25,26,27]. The easy cultivation of algae and cyanobacteria, which uses wastewater as an alternative culture medium and non-agricultural land for installing open reactors or the macroalgae collection from offshore, makes them viable candidates for various applications. In our case, we propose their use as a promising alternative for researching and producing biocontrol agents against *Fusarium* spp. In addition, in the cultivation of microalgae and cyanobacteria, abiotic stress induced by changes in pH, temperature, light intensity, and agitation in the photobioreactor influences the production of secondary metabolites, which may exhibit antifungal activity [28,29]. Several studies report antifungal activity in various macroalgae, microalgae, and cyanobacteria [30]. One study found that methanol extracts, which are rich in phenolic compounds, from *Ulva lactuca*, *Sargassum dentifolium*, and *Gracilaria* sp. effectively inhibited *F. oxysporum* growth in vitro. Additionally, a greenhouse trial with *S. dentifolium* showed a 40.8% reduction in *Fusarium* disease in tomatoes [31]. On the other hand, the microalgae *Chlorella vulgaris*, *Dunaliella salina*, *Scenedesmus obliquus*, *Tetraselmis suecica*, and *Nannochloropsis oculata* have also been studied for their in vitro antifungal activity against *Fusarium* spp. Regarding cyanobacteria, although they are the least reported, studies have found that conclude that the genera *Nostoc*, *Spirulina*, *Anabaena*, *Westiellopsis*, *Hapalosiphon hibernicus*, and *Microcystis* have antifungal activity [32,33].

Among algae and cyanobacterial metabolites with antifungal activity against *Fusarium* spp., fatty acids represent a promising area of study since their efficacy in inhibiting this pathogen has been demonstrated [34]. Algae and cyanobacteria synthesize various fatty acids, including polyunsaturated, medium-chain, and short-chain fatty acids. These molecules have been reported to possess antimicrobial, insecticidal, and antifungal properties, with a minimum inhibitory concentration (MIC) of 600 μg·mL^−1^ against *C. albicans* [35,36,37]. Studies indicate that fatty acids from algae and cyanobacteria can exert their antifungal activity against fungi and *Fusarium* spp. through several mechanisms, such as inhibition of fungal spore germination, destruction of the fungal cell wall, inhibition of lipid synthesis, induction of defense compounds in plants, and activation of elicitors and signaling compounds [38,39].

Despite extensive research on the antifungal potential of algae and cyanobacteria, there remains a critical gap in the literature regarding the specific efficacy of fatty acids, which are derived from these organisms, against the phytopathogenic fungus *Fusarium* spp. This review addresses that gap by comprehensively analyzing the antifungal effects of polar and non-polar algae extracts against *Fusarium*. The analysis will encompass macroalgae, microalgae, and cyanobacteria, with a particular emphasis on elucidating the role of fatty acids in this context and exploring whether the extracts exhibit biostimulant and/or fertilizing properties.

## 2. Materials and Methods

For this literature review, an exhaustive search was conducted in the PubMed (https://pubmed.ncbi.nlm.nih.gov), Crossref (https://www.crossref.org), and Scopus (https://www.scopus.com) databases before 29 November 2024. The initial selection of literature was based on the following keywords: #1: “Fusarium” or “phytopathogen”; #2: “macroalgae”, “microalgae”, “cyanobacteria*”, “natural extract”, or “antipathogen”; #3: “fatty acids”, “peptides”, or “metabolites”. The publication date ranged from 1 January 2014 to 29 November 2024. The search results were integrated using a Boolean search’s logical “AND” operator. Subsequently, the retrieved articles were filtered to exclude those of limited relevance, prioritizing studies specifically addressing the use of algal metabolites in developing valuable antifungal products, with a clear focus on *Fusarium* spp. and algae. This filtering process was conducted by analyzing each article’s title, keywords, and abstract.

## 3. *Fusarium*: Impact and Challenges in Agriculture

### 3.1. Morphology Characteristics

**Figure 1:**
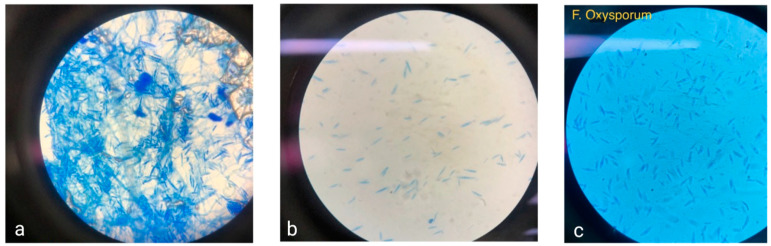
(**a**,**b**) Microscopic views of Mexican corn isolates. (**c**) *F. oxysporum* at 40×.

*Fusarium* is a genus of ascomycetous fungi notable for the loss of sexual reproduction. This genus includes around 70 recognized species, identified through a polyphasic approach, and around 300 putative species. Morphologically, *Fusarium*. are characterized by conidia formation in chains on different types of conidiophores. These include macroconidia (3–5 cells, curved) that are produced as asexual reproductive structures and microconidia (1–2 cells) of straight or slightly curved ovoid shape and chlamydospores [40]. *Fusarium* hyphae are hyaline and septate, with a diameter of 3 to 8 μm (Figure 1a,b). The *Fusarium* cell wall consists of an outer layer of glycoproteins, mannose, and melanin, while the inner layer is composed of β-(1-3)-glucans, β-(1-6)-glucans, and chitin [41]. The cellular membrane of *Fusarium* is primarily made up of phospholipids and ergosterol [42]. The *Fusarium* genus is distinguished by its cottony mycelium, often displaying characteristic pigmentation in shades of violet, pink, or yellow. This pigmentation varies among species; for instance, *F. solani* may exhibit red, pink, brown, or gray hues, *Fusarium verticillioides* typically appears white with yellow and purple tones, and *F. oxysporum* (Figure 1c) presents as cottony white with a yellow center [43].

*Fusarium* spp., in addition to its distinct morphological characteristics, exhibits remarkable adaptability to a wide range of environmental conditions. These fungi predominantly colonize warm, sandy soils where they can persist over long periods, with optimal growth occurring at temperatures between 20 and 32 °C. *Fusarium* spp. [44]. can be part of the typical soil crop microflora, contributing to a healthy ecosystem. However, some species act as plant pathogens, causing diseases in fruits and cereals crops, such as tomato, wheat, and corn. *Fusarium* spp. frequently inhabit roots and plant debris, acting as saprophytes [45]. Soil pH also plays a role in modulating *Fusarium* spp. growth; under stress conditions, they may produce various mycotoxins, such as fusaric acid and fumonisins, which serve as valuable identifiers for species differentiation and crop impact assessment [46]. Globally, *Fusarium* spp. ranks among the most critical phytopathogens, contributing to a wide array of diseases across both agricultural and horticultural crops.

### 3.2. General Description of Fusarium spp. Infection Mechanism as a Plant Pathogen

*Fusarium* spp. can infect plants at any stage of development, leading to seedling, root, crown rot, stem, and even tassel rot. The phytopathogenicity of *Fusarium* spp. is caused by a combination of mechanisms [47]. Among them, the fungus synthesizes enzymes like pectinase, cellulase, protease, and lipase, which degrade the plant cell walls to allow the fungus to invade [43]. Furthermore, *Fusarium* spp. produces toxins such as fusaric acid, fumonisins, and ochratoxin A. These toxins are highly toxic to both plants and animals, contributing to disease symptoms and potential health problems if consumed [4]. Additionally, *Fusarium* spp. utilizes G protein to regulate their response to environmental stress. These proteins allow the fungus to adapt to different crop conditions, making it a more persistent threat [48].

*Fusarium* infection develops in stages. In the first stage, the hyphae of the fungus adhere to the surface of the plant roots, then mechanically penetrate the plant tissue, thanks to the pressure of the hyphae and secreted enzymes, which degrade the defense barriers of the cell walls [49]. The fungus must tolerate the plant antifungal compounds and inhibit its defense response by secreting phytotoxins. Once the fungus colonizes the plant, its mycelium spreads intracellularly until it reaches the xylem of stems and leaves, absorbing the nutrients necessary for its development [50]. This fungal pathogen disrupts the plant vascular system, leading to wilting, chlorosis (yellowing), and ultimately plant death. Vascular wilt specifically targets the vascular system, hindering the transport of water and essential nutrients [51]. Colonization is rapid due to the formation of microconidia within the xylem, where the spores germinate, and the germ tubes pass through the xylem cells, subsequently forming hyphae, conidiophores, and conidia [40]. The mycelium of the fungus accumulates in the vascular tissues, while the parenchyma cells multiply as a plant defense response, secreting toxins that compress the vessels. The characteristic wilting of *Fusarium* spp. diseases results from water stress due to the limitation of the functioning of the vessels, which can generate low productivity or even the death of the plant. This invasion also causes root and stem rot [52].

### 3.3. Damage to Most Important Crops: Diseases Caused by Fusarium spp.

Fungal diseases caused by *Fusarium* species present a major global challenge to agriculture. *Fusarium* spp. infects a wide range of crops, impacting not only cereals and legumes but also causing root and stem rot in fruits and vegetables [53,54]. Crops like tomatoes, bananas, and cotton are particularly susceptible to vascular wilt. Similarly, maize is vulnerable to *Fusarium* spp.-mediated ear rot, which leads to substantial reductions in both grain yield and quality [6,55], thus compromising food production and quality. Two of the most common *Fusarium*-related diseases affecting cereal crops worldwide are *Fusarium* head blight (FHB) and *Fusarium* crown rot (FCR). FHB, which primarily targets wheat, corn, and barley, causes rotting of the grain head, leading to significant reductions in both grain yield and quality [56]. Additionally, infections by some *Fusarium* result in the production of fumonisins, a group of mycotoxins in grains that are highly toxic to humans and animals [4]. Table 1 provides an overview of *Fusarium* diseases, including FHB and FCR, and highlights their detection technique. In the United States, a leading global wheat producer, FHB can cause yield losses exceeding 30%, involving at least 17 *Fusarium* spp. Infected grains often wither and discolor prematurely, leading to spike death. Additionally, FHB contamination with mycotoxins presents significant storage risks as these toxins can persist for years, reducing grain market value. Recent U.S. market discounts have ranged from USD 1.84 to 3.67 per ton for every 0.5 ppm of mycotoxin detected in grains [57].

On the other hand, FCR can persist in wheat crops in arid and semi-arid regions, as fungal hyphae survive in plant residues for up to three years under suitable environmental conditions. The infection begins at the roots as the plant grows and subsequently spreads throughout the plant. FCR, caused by *Fusarium pseudograminearum*, *F. oxysporum* and *F. culmorum*, either individually or together, affects the crown and basal stem tissues, hindering water transport from the roots to the canopy [58]. Severe infection in basal tissues leads to premature senescence of the heads, known as whiteheads. FCR typically progresses from a chronic, low-impact disease to an acute, destructive one during drought stress around or after anthesis [59].

**Figure 2:**
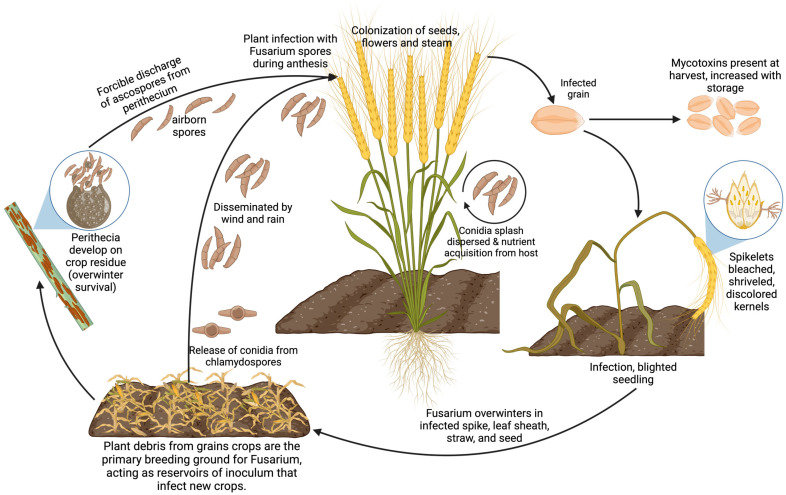
Fusarium life cycle: from soil to plant wilting.

The development of *Fusarium* infections is often aggravated by agricultural practices such as inadequate tillage in intensive cropping systems and the improper application of fungicides, combined with climatic conditions that enhance the pathogen’s survival during its saprophytic phase in crop residues [60] (Figure 2). Moreover, the variability in pathogenicity, toxicity, and fungicide sensitivity across different *Fusarium* spp. strains poses significant challenges to controlling efforts, thereby fueling scientific interest in the discovery of broad-spectrum biocontrol agents [7].

**Table 1:** Pathogenic *Fusarium* species, their hosts, associated diseases, and detection methods.

Pathogen	Disease Caused	Host/Plant Part	Detection Technique	Reference
*Fusarium* spp.	Wheat crown rot	Wheat/Wheatears	PCR-based methods	[61]
*F. verticillioides*	Fumonisins	Corn	Immunoassay kits, PCR-ELISA	[62]
*F. oxysporum* *Fusarium cubense*	Panama disease (*Fusarium* wilt)	Banana/Roots	Raman spectroscopic fingerprints	[63]
*F. solani*	Coffee corky root	Coffee seedlings	Isolation and identification of fungal structures, species-specific PCR	[64]
*F. oxysporum*	Fusarium wilt	Melon	Loop-mediated isothermal amplification (LAMP), PCR	[65]
*F. equiseti*	Potato dry rot	Potato/Tubers	Isolation and identification of fungal structures, PCR	[66]
*F. equiseti*	Foliar disease	Lettuce plants	Real-time PCR, digital PCR	[67]
*Fusarium proliferatum*	Clove rot	Garlic	Near-infrared spectroscopy (NIRS)	[68]
*Fusarium sambucinum*	*Fusarium* canker	*Humulus lupulus*	Amplified polymorphic DNA PCR assay	[69]
*Fusarium virguliforme*	Soybean Sudden Death Syndrome	Soybean	TaqMan qPCR assay based on the ribosomal DNA (rDNA) intergenic spacer	[70]

ELISA (Enzyme-Linked Immunosorbent Assay), DNA (Deoxyribonucleic Acid) analysis, TaqMan (Taq Polymerase-based Fluorescent Probe System), qPCR (Quantitative Polymerase Chain Reaction), rDNA (Recombinant DNA), PCR (Polymerase Chain Reaction), and LAMP (Loop-Mediated Isothermal Amplification).

### 3.4. Management Strategies for Diseases Caused by Fusarium spp.

Managing *Fusarium* spp. diseases requires a comprehensive approach that integrates multiple strategies. Implementing effective cultural practices can significantly reduce soil pathogen populations and minimize the risk of disease. Genetically resistant crops are a cornerstone of prevention [71], with the selection of varieties containing specific resistance genes against pathogen races being key to this approach. Additional cultural strategies include crop rotation with non-host species of *Fusarium*, proper tillage to promote residue decomposition, and the removal of infected plant material at the end of the harvest cycle. These practices are essential for effective disease management [72].

Nonetheless, the control of diseases caused by *Fusarium* spp. still relies heavily on chemical fungicides, which pose environmental and public health risks and contribute to fungal resistance. In this context, searching for natural alternatives and sustainable control methods is important. Biocontrol strategies against *Fusarium* spp. involve both direct and indirect mechanisms. Direct mechanisms include the production of antifungal compounds and secondary metabolites that inhibit the growth of the pathogen. In contrast, indirect mechanisms involve competition for nutrients and colonization space, which limits the pathogen’s ability to thrive. Understanding these metabolic pathways and biocontrol mechanisms provides valuable insights for the development of novel and more effective biocontrol agents [40]. The development of new biocontrol methods for combating diseases caused by *Fusarium* spp. has increasingly focused on obtaining antifungal compounds or secondary metabolites from plants and microorganisms. In this context, a study of 88 actinomycete isolates identified *Streptomyces violaceusniger* as a promising biocontrol agent. This strain demonstrated a potent inhibition efficiency of 60.46% against *F. oxysporum* by disrupting the membrane integrity and ultrastructure of fungal cells. Notably, its secondary metabolites, 5-hydroxymethyl-2-furancarboxaldehyde and n-hexadecenoic acid, have been identified as antifungal compounds. The former has been previously described as an intermediate in agricultural chemicals, while the latter exhibits cytotoxic properties, thus highlighting their potential for the effective management of diseases caused by *Fusarium* spp. [73]. Li et al. (2021) demonstrated that methanolic extracts of *Streptomyces* sp. exhibited antifungal activity against *F. oxysporum*, inhibiting 86.45% of mycelial growth and reducing the disease index in banana seedlings. This effect was linked to membrane disruption and ultrastructural damage in *Fusarium* sp. cells, which lead to nucleic acid release and increased extracellular conductivity, indicating plasma membrane damage [74].

A previous study identified several phenolic acids as bioactive compounds with antifungal properties achieving up to 88.9% of inhibition against *F. oxysporum*. Notable compounds included p-hydroxybenzoic acid, benzoic acid, gallic acid, rosmarinic acid, and o-coumaric acid, which were found in extracts of *Conium maculatum* (leaves), *Acacia salign* (bark), *Schinus terebinthifolius* (wood), and *Ficus eriobotryoides* (leaves) [75]. Alkaloids, including piperaduncin, asebogenin, and methyllinderatin, were found in *Piper aduncum* extract with a mycelial growth inhibition of 95.26%. Berberine has also been shown to exhibit antifungal properties by inducing the accumulation of reactive oxygen species, leading to cell death, with EC_50_ values of 0.065 μg·mL^−1^ against *R. solani* [76,77]. Among the metabolites that have shown excellent results are essential oils, such as tea tree oil, oregano oil, and garlic and turmeric extracts. However, the primary challenge in applying natural-origin compounds lies in scaling up their production to enable the development of large-scale methods for bioactive compound synthesis. A study examined the antifungal activities and fatty acid composition of Jatropha oils. The oils were tested at 1% and 2% concentrations against *Fusarium solani* strains isolated from tomato plants. The results showed that both concentrations inhibited fungal growth and reduced sporulation, with a maximum inhibition of 72.7%. Gas chromatography/mass spectrometry (GC-MS) analysis revealed that both oils were predominantly unsaturated, with the main fatty acids being vaccenic, nonadecanoic, linoleic, palmitic, myristic, and erucic acids [78]. In addition to essential oils, fatty acids in *A. jensenii* and *L. rhamnosus* (3-(4-hydroxyphenyl) propionic acid) also exhibit higher antifungal activity. Derivatives of hydroxydecanoic acid inhibit the growth of *R. mucilaginosa* at 10 μg·mL^−1^, contributing to antifungal activity. These findings suggest that the viscosity and reactivity of fatty acids facilitate their integration into fungal cell membranes, promoting membrane disruption through their lipolytic activity [79].

Certain microorganisms act as antagonists to *Fusarium* spp. through mechanisms that combine antifungal activity with plant growth promotion. A previous study showed that *Aspergillus fumigatus* and *Rhizopus oryzae*, both plant growth promoters, exhibited antifungal activity against *F. oxysporum*, the cause of *Fusarium* spp. wilt in tomatoes. The study reports that the MIC of *R. oryzae* extract was 0.5 m mL^−1^, while the MIC of *A. fumigatus* extract was 1 mg·mL^−1^. *In vivo* assays demonstrated disease severity reductions of 12.5% and 37.5% for *A. fumigatus* and *R. oryzae*, respectively, with protection levels of 86.35% and 59.06% [80]. Another study by Rojas et al. (2020) [81] reported that wheat endophytic fungi, such as *Anthracocystis floccosa* and *Penicillium olsonii*, exhibit potential as biocontrol agents against *Fusarium* spp. These fungi colonize plant tissues asymptomatically while occupying the same biological niche as the pathogens. Similarly, research by Kemp et al. (2020) [82] demonstrated that the endophytic fungus *Sarocladium zeae* stimulates the secretion of defense-related hormones in wheat plants, effectively activating their innate defense mechanisms. This enhanced defense response in seedlings was associated with a 57.9% reduction in disease severity and a 61.2% decrease in the accumulation of mycotoxins such as deoxynivalenol. Antagonistic microorganisms have been effectively utilized as a biological control strategy against *Fusarium* spp., which employs various mechanisms such as nutrient competition, production of antifungal metabolites, interaction through structural and biochemical mechanisms, and stimulation of plant defenses to enhance stress tolerance [83]. This approach represents a sustainable and environmentally friendly alternative to traditional *Fusarium* sp. management. Currently, a wide range of products is available on the market, most of which are derived from bacteria (Table 2) (all websites were accessed on 29 November 2024). Table 2 presents three commercial biofungicides containing *Bacillus* spp. (Gram-positive) as active ingredients: Serenade Garden Disease Control, Companion^®^ Biofungicide, and Trilogy^®^ Biofungicide. The mode of action involves plant root colonization and the production of antifungal compounds. While the specific antifungal metabolites are not listed, *Bacillus* spp. are known to produce volatile organic compounds (3-methyl-1-butanol, 2-methyl-1-butanol, butane-1-methoxy-3-methyl, 2-hydroxy-3-pentanone, 3-methyl-2-pentane, and methanethiol) [84], as well as lipopeptides like surfactin, iturin, and fengycin, which show antifungal activity against *Fusarium* spp. [85,86]

Additionally, Table 2 lists RootShield^®^ 10G Biofungicide, which contains *Trichoderma harzianum* as the active ingredient. While the specific antifungal metabolites are not specified, *Trichoderma* spp. are known to produce volatile organic compounds such as butenolides [87], phenolic compounds like flavonoids and catechins [88], and saturated and polyunsaturated fatty acids (pentadecanoic acid, palmitic acid, methyl linolelaidate, linoleic acid, and 9,17-octadecadienal) [89], all with antifungal activity against *Fusarium* spp.

Although microorganisms and terrestrial plant extracts have proven to be effective, antifungals, macroalgae, microalgae, and cyanobacteria emerge as even more promising organisms due to their abundance, rapid growth, and unique bioactive compounds. These include their ability to grow in wastewater or saline environments, their non-competition with arable land, their CO_2_ capture capabilities, and the lower cost of culture media. Additionally, their biochemical composition can be easily controlled by optimizing cultivation parameters. These organisms represent a valuable source of bioactive compounds and secondary metabolites with diverse biological activities, such as antifungal, antibacterial, antiviral, anticancer, and antioxidant properties [90,91]. Numerous studies highlight the potential of their extracts and compounds for the development of pharmaceutical, nutraceutical, and agrochemical products [92,93]. A key advantage of using these organisms is their biodiversity as each species possesses a unique metabolic profile, thus increasing the likelihood of discovering new compounds with antifungal activity. Furthermore, macroalgae, microalgae, and cyanobacteria can be easily cultivated with fewer resources than terrestrial plants, which makes them efficient producers of secondary metabolites at high rates [94]. Algal and cyanobacterial compounds have shown potential for controlling diseases like *Fusarium* spp. through both direct antagonistic activity and the induction of plant defense. Recent research suggests *Ascophyllum nodosum* extract use can reduce abiotic stress-induced toxicities in plants and offers an eco-friendly alternative to synthetic fungicides. However, further research is needed to optimize their use and effectiveness in real-world conditions, particularly in controlling fungal pathogens like *Fusarium* spp. under practical cultivation conditions [95].

## 4. Algae and Cyanobacteria as Sources of Antifungal Compounds

Algae refers to a large group of unicellular or multicellular plant-like organisms that range in size from 1 µm to the giant kelp, which can grow up to 60 m in length [96]. These organisms are aquatic, oxygenic, phototrophic, and photosynthetic [97]. There are over 55,000 species and around 10 million strains, which can be classified into nine main divisions based on their size, pigmentation, structure, development rate, and cell wall phytochemicals [97]. Macroalgae, commonly known as seaweed, are multicellular and macroscopic. They are plant-like organisms but lack functional tissues [96]. On the other hand, microalgae are microscopic, unicellular forms of algae that exist as individual cells or colonies in both natural and marine waters [98]. They typically store 40–80% lipids (dry weight) and 6–75% proteins (dry weight) [99]. Their simple unicellular structure allows for faster division, leading to high reproduction and growth rates. Cyanobacteria (often referred to as blue-green algae) are an ancient group of prokaryotic, single-celled organisms [100]. They can form colonies that appear as filaments, sheets, or short, slimy biofilms [101]. Algae represent a diverse group that includes macroalgae, microalgae, and cyanobacteria, with the main differences lying in their size and evolutionary adaptations.

Macroalgae, microalgae, and cyanobacteria all contain chlorophyll, which enables them to absorb sunlight and perform photosynthesis [97]. In macroalgae, photosynthesis occurs in the stroma of chloroplasts, while in cyanobacteria, it takes place within thylakoids [102]. During the light-dependent phase, photons are absorbed by pigments like phycobilins, passing through Photosystems II and I, which leads to the production of ATP and NADPH [102]. These molecules drive the Calvin–Benson–Bassham cycle, where carbon is fixed into glucose, which enters glycolysis [102]. Other pathways, such as the oxidative pentose phosphate pathway, contribute to secondary metabolism, ultimately producing bioactive compounds [97,102,103]. These bioactive compounds, including antifungal agents, make algae and cyanobacteria promising sources of natural antifungal alternatives.

Macroalgae, microalgae, and cyanobacteria, predominantly found in marine environments, are subject to various biotic and abiotic factors that influence their metabolism. Biotically, these organisms are susceptible to predators, which prompts them to synthesize and release extracellular molecules and secondary metabolites as a defense mechanism [104]. This phenomenon, known as allelopathy, involves the release of bioactive molecules for inhibiting the growth or activity of other organisms, including microorganisms, plants, and insects [105]. In algae, these allelochemicals include fatty acids and their derivatives, phenolic compounds, alkaloids, peptides, and volatile organic compounds, each synthesized via specific metabolic pathways. Fatty acids and their derivatives are produced from Acetyl-CoA through pathways that yield monounsaturated and polyunsaturated fatty acids, with derivatives formed via oxidative transformations [106]. Algae phenolic compounds, derived from the phenylpropanoid pathway using phenylalanine and tyrosine as precursors, include substances such as gallic acid, cinnamic acid, rosmarinic acid, and quercetin. These compounds modify the structure of the fungal cell membrane, resulting in the loss of K^+^, a reduction in ATP, and the disruption of essential enzymatic processes required for fungal survival [107]. Terpenoids, such as those in *Dictyopteris polypodioides*, induce stress in fungi by triggering calcium bursts within cells, damaging cell membranes, and exhibiting strong antifungal activity, particularly against the *Fusarium* species [95]. Alkaloids, characterized by nitrogen-containing heterocyclic structures, originate from amino acids [26]. Peptides are synthesized through non-ribosomal peptide synthesis [108]. Lastly, volatile organic compounds, such as terpenoids, hydrocarbons, and ketones, are released when algae are attacked by predators or undergo senescence [109].

The chemical and structural properties of these allelochemicals enable them to inhibit microorganisms, including fungi. These bioactive molecules from algae exhibit bio-fungistatic properties and, in some cases, bio-fungicidal activity against phytopathogenic fungi such as *F. oxysporum*, *Phytophthora capsica*, *Alternaria alternata*, *Rhizoctonia*, *Rosellinia*, and *Sclerotinia*. The antifungal activity of algal extracts shows a linear relationship with their concentration and evaluated parameters such as mycelial growth and dry weight [3]. Cyanobacteria such as *Nostoc*, *Anabaena*, *Microcystis*, and *Scytonema* have the unique ability to synthesize bioactive compounds with antifungal and antibacterial properties. These metabolites effectively control plant diseases caused by soil pathogens like *Fusarium* and *Rhizoctonia* in a highly selective manner, targeting pathogens without harming beneficial organisms in the ecosystem. Unlike conventional chemical pesticides, which can have broad, non-selective effects, these metabolites provide a more targeted and environmentally friendly approach to pest management. However, some cyanobacteria produce cytotoxic compounds that may adversely affect treated plants [110]. Cyanoabacteria produce a cyanotoxins as cytotoxins like cylindrospermopsin [111] can present possible antifungal activity against *Fusarium* spp. Considering that crop plants are vulnerable to over 10,000 species of phytopathogenic fungi, allelochemicals from algae represent a valuable source of antifungal compounds. Their mechanisms of action often involve targeting fungal cell walls and membranes. For example, fatty acids penetrate the chitin cell wall and interact with the ergosterol-rich cell membrane, increasing its fluidity and inducing conformational changes that lead to cell degradation [34]. Volatile organic compounds disrupt membrane integrity by peroxidizing membrane lipids [112]. Phenolic compounds interfere with cellular respiration by altering cytochrome pathways, while cyclic peptides form pores in the fungal membrane due to electrostatic interactions between their terminal amino acids and polysaccharides in the cell wall [113,114]. Alkaloids compromise fungal cell wall morphology and membrane integrity [115].

**Figure 3:**
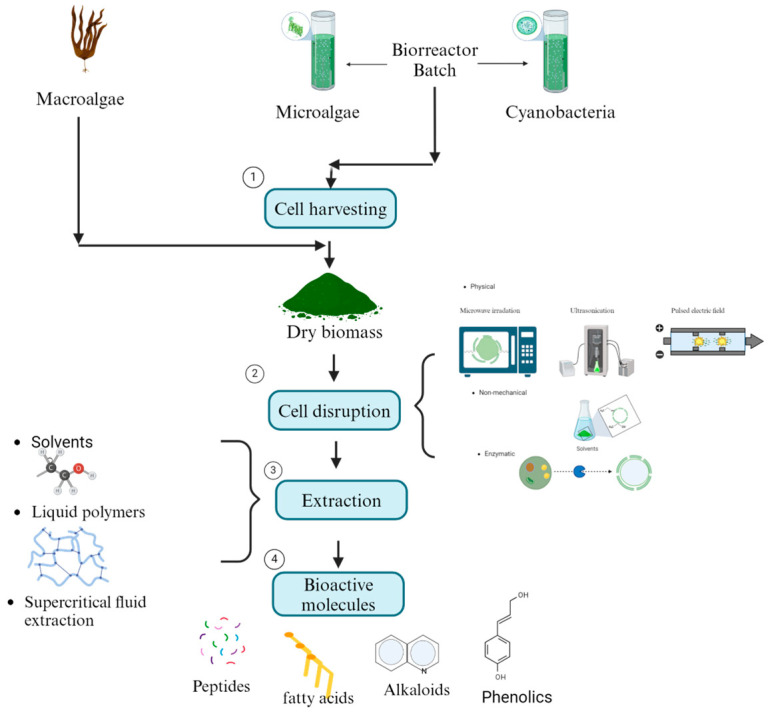
Diagram with the steps of primary recovery of bioactive molecules of macro-, micro-, and cyanobacteria biomass.

Algal bioactive molecules can be utilized in two primary ways. The first involves using living microalgae and cyanobacteria cells, which naturally release allelochemicals to inhibit phytopathogenic fungi [116]. However, this approach has been underexplored in soil environments, where the survival and efficacy of microalgae and cyanobacteria remain unclear [117]. The second, more commonly studied method involves lysing macroalgae, microalgae, and cyanobacteria cells to release their bioactive compounds. This is achieved using mechanical methods, such as microwave irradiation, ultrasonication, pulsed electric fields, and thermal treatments, or non-mechanical methods involving chemical solvents, acids, alkalis, detergents, and enzymatic treatments [118]. The bioactive molecules are then extracted using techniques such as solvent extraction with polar and non-polar solvents (e.g., diethyl ether, chloroform, ethyl acetate, acetone, ethanol, methanol, and water) or emerging bio-based solvents like ionic liquids and liquid polymers [119]. Supercritical fluid extraction (SFE) is another well-established technique that allows simultaneous extraction and separation by optimizing parameters such as temperature, pressure, flow rate, and processing time [120]. These extraction steps are essential for isolating bioactive molecules efficiently (Figure 3). Finally, the antifungal activity of crude or fractionated extracts is assessed using diffusion and dilution assays [121]. 

Due to the focus of this review on the phytopathogen *Fusarium* spp., we summarize the research presented in Table 3, which reports on the antifungal activity of crude biomass extracts from macroalgae, microalgae, and cyanobacteria against *Fusarium* spp. Firstly, regarding cyanobacteria strains such as *Spirulina* sp., Scaglioni et al. (2019) [122] conducted an antifungal assay using *Spirulina* sp. aqueous extract by agar dilution at 25 °C for 7 days against *F. graminearum* and *F. meridionale* and they reported a mycelial growth rate as low as 0.21 cm·day^−1^, using water extracts. Arnhold et al. (2014) [123] used the same method and cyanobacterium but tested at 35–40 °C; the authors observed mycelial growth rates ranging from 0.1 cm·day^−1^ to 0.45 cm·day^−1^ for *F. meridionale* and *F. graminearum* using methanolic extracts. Both studies identified phenolic acids, including chlorogenic acid, protocatechuic acid, gallic acid, and caffeic acid, as the metabolites responsible for the antifungal activity. These phenolic compounds were found in both aqueous and methanolic extracts, with high-performance liquid chromatography coupled mass spectrometry (HPLC/MS) used to separate and identify them. Additionally, *Nostoc calcicola* and *Nostoc carneum*, as reported by El-Sheekh et al. (2020) [38] and Farghl and El-Sheekh (2019) [124], exhibited inhibition zones of 16 mm and 160 mm, respectively, when using methanolic and ethyl acetate extracts. GC-MS analysis identified aromatic carboxylic acids and terpenoids as the active metabolites in these extracts.

In the case of macroalgae, Mostafa et al. (2022) reported a mycelial growth diameter of 41, 49, and 45 mm for the methanolic extracts of *S. dentifolium*, *G. compressa*, and *U. lactuca*, respectively, at a concentration of 20 µg·mL against *F. oxysporum* f. sp. *lycopersici*. The metabolites identified in the extract by high-performance liquid chromatography/ultraviolet-visible (HPLC/UV-Vis) were phenolics and included phloroglucinol, gallic acid, and vanillic acid [30]. On the other hand, Mohy El-Din and Mohyeldin (2018) reported inhibition zones of 19, 9, 19, and 11 mm against *F. solani* with the methanolic extracts of *C. sinuosa*, *P. pavonia*, *C. barbata*, and *S. vulgare* and with metabolites such as aromatic carboxylic acids and volatiles identified by GC/MS [126].

For microalgae, Senousy et al. (2022) and Scaglioni et al. (2019) identified phenolic acids such as chlorogenic acid, gallic acid, and protocatechuic acid as metabolites with antifungal activity in methanolic extracts of the biomass of *Dunaliella* sp., *C. sorokiniana*, and *Nannochloropsis* sp. In the case of *Dunaliella* sp. and *C. sorokiniana*, the extracts showed 82% and 40% inhibition, respectively, against *F. solani*. The methanolic extract of *Nannochloropsis* sp. exhibited a mycelial growth rate lower than 0.32 cm·day^−1^ against *F. asiaticum*, *F. graminearum*, and *F. meridionale* [122,127]. Additionally, Alallaf et al. (2021) reported that the hexane extract of *N. arenaria* biomass showed 22% inhibition at 6000 µg·mL^−1^, with a minimum inhibitory concentration (MIC) against *F. oxysporum*. GC-MS analysis identified aromatic carboxylic acids and sterols such as di-n-octyl phthalate and beta-sitosterol as the active metabolites [128].

### Role of Algae Fatty Acids as Antifungal

The fatty acids from macroalgae, microalgae, and cyanobacteria receive special attention in this review because, when researchers analyze the metabolite profiles of bioactive molecules using GC-MS, esterified fatty acids, whether unsaturated, polyunsaturated, or a combination of both, are often among the most abundant compounds identified. This makes it interesting to discuss how these fatty acids are synthesized in macroalgae, microalgae, and cyanobacteria in general and how these biomolecules exhibit antifungal activity against *Fusarium* spp. This includes exploring the mode of antifungal action, which is discussed in the following section.

At the end of glycolysis, pyruvate is converted into Acetyl-CoA, which begins the synthesis of free fatty acids. Acetyl-CoA is first converted into Malonyl-CoA, which is then transferred to acyl carrier proteins (ACP) and undergoes multiple enzymatic reactions to form fatty-acyl-ACP [106]. Enzymes like β-ketoacyl-ACP synthase and β-ketoacyl-ACP reductase participate in elongation and desaturation cycles, producing fatty acids ranging from C_4_ to C_18_. Finally, Acyl-ACP thioesterase releases free fatty acids, which are essential for the production of bioactive compounds, including those with antifungal properties [129,130]. Polyunsaturated fatty acids (PUFAs), such as oleic acid (C18:1), linoleic acid (C18:2), and arachidonic acid (C20:4), are produced from C_16_–C_18_ fatty acids through desaturation by ${∆}^{9}$ and ${∆}^{12}$ desaturases [131,132]. Even though Acyl-ACP thioesterase is absent, the presence of free fatty acids in the cytoplasm of algae is due to a lipid remodeling or lipid degradation process in which free fatty acids are released from the membrane by lipase and are subsequently esterified with the thiol of acyl carrier protein by acyl–acyl carrier protein synthases [130]. These PUFAs, along with their derivatives, such as fatty alcohols and alkanes, have demonstrated antifungal activity. Notably, the extract exhibited an MIC_50_ of 50 µg·mL^−1^ against various pathogens, including *Fusarium* spp.

The fatty acids synthesized by microalgae and cyanobacteria can exhibit antifungal activity when released into the extracellular matrix through the action of lipase enzymes. Alternatively, free fatty acids can be extracted from biomass using cell lysis techniques and solvent extraction, with polar solvents like methanol and ethanol or non-polar solvents like chloroform [3]. In this review, we will focus on fatty acids extracted from dry biomass using polar solvents by specifically examining their effects on the mycelium inhibition zone (mm), percentage of inhibition, and minimal inhibitory concentration (µg mL^−1^).

**Figure 4:**
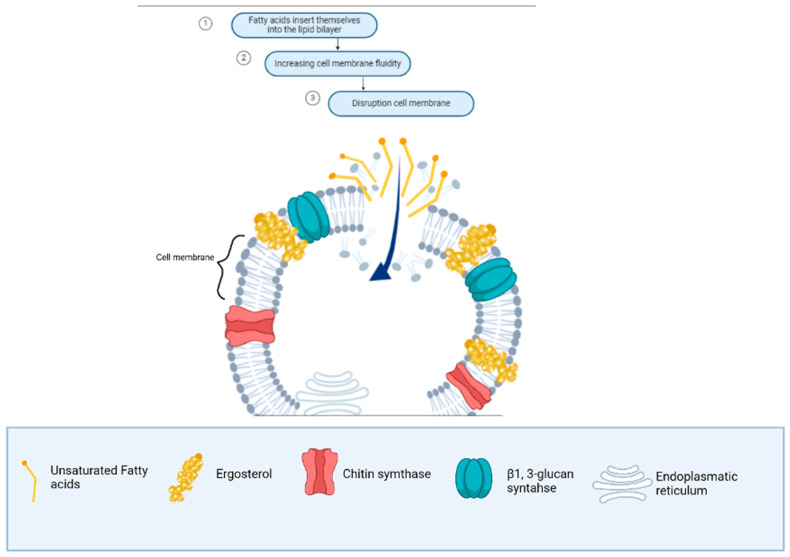
Antifungal mechanism of unsaturated fatty acids from macro-, micro-, and cyanobacteria in the cell membrane of *Fusarium* spp.

Firstly, it is important to understand the antifungal mechanism of fatty acids as carboxylic acid molecules. When these fatty acids pass through the cell wall, they interact with the ergosterol-rich membrane, disrupting its stability and cellular functions [133]. Figure 4 illustrates the sequential steps involved: increased membrane fluidity (dependent on sterol content) leads to membrane expansion, causes conformational changes in membrane proteins, and ultimately results in cytoplasmic disintegration. However, it is important to consider that the effectiveness of the antifungal activity of fatty acids depends on the sterol concentration in the fungal membrane. At low sterol concentrations, fatty acids are more effective, while at high sterol concentrations, fungi may exhibit resistance [34]. The structure of the fatty acids, including the presence of double bonds (saturated or polyunsaturated) or hydroxy groups, also influences their interaction with the cell membrane [134].

The hydrophobic groups of saturated fatty acids are involved in antifungal activity by interacting with the cell membrane, which leads to its disintegration due to changes in intracellular hydrostatic turgor pressure [34]. For example, lauric acid (C12:0) at concentrations of 600–1200 mg·L^−1^ and capric acid (C10:0) at 300–1200 mg·L^−1^ exhibit antifungal effects [135], while Bhattacharyya et al. (2020) report that a 0.2% p/v concentration is effective in disrupting the granular cytoplasm [136]. Structurally, tanikoloide is a saturated fatty acid, with a lactone group attached to a hydroxy methylene group; it is isolated from the lipophilic extract of the cyanobacterium *Lyngbya majuscula*, which also demonstrates antifungal properties [137]. As shown in Table 4, El-Sheekh et al. (2022) reported the mass spectrum of two saturated fatty acids, 12,15-octadecadienoic acid methyl ester and palmitic acid, which are isolated from a methanolic extract of *N. calcicola* and exhibited a 16 mm inhibition zone against *F. oxysporum* [138]. Similarly, a study identified three saturated fatty acids—tetradecanoic acid methyl ester, hexadecanoic acid methyl ester, and eicosanoic acid methyl ester—from an ethyl acetate extract of the *Nostoc carneum* pellet, which showed a mycelial inhibition of 3–160 mm against *F. oxysporum* at a concentration of 300,000 µg·L^−1^ [124]. Additionally, Perveen and Alwathnani (2013) reported that compounds such as 3-chloropropionic acid, heptadecyl ester, 1,2-benzene dicarboxylic acid bis (2-ethoxyethyl) ester, and bromoacetic acid, pentadecyl ester—extracted from a methanol:acetone:diethyl ether extract of *S. platensis* biomass—demonstrated strong antifungal activity against *F. Oxysporum* [125].

As shown in Table 4, El-Sheekh et al. (2020) reported saturated fatty acids, including hexadecanoic acid methyl ester, 9-octadecenoic acid methyl ester, tetradecanoic acid methyl ester, and cis-11-eicosenoic acid methyl ester, from methanol and acetone extracts of *Cystoseira myrica*, *Padina boergesenii*, and *Sargassum cinereum*. These exhibited inhibition zones of 15 mm, 13 mm, and 14 mm, respectively, against *F. oxysporum* [38]. Similarly, Mohamed and Saber (2019) reported palmitic acid, myristic acid, and stearic acid as saturated fatty acids, with a MIC of 6 µg·mL^−1^ in chloroform extracts from *Hemidiscus cuneiformis*, showing a 16 mm inhibition zone against *F. oxysporum* [139]. Furthermore, Mohy El-Din and Mohyeldin (2018) identified palmitic acid and myristic acid in *C. sinuosa*, *P. pavonia*, *C. barbata*, and *S. Vulgare* methanol extracts, with inhibition zones of 19 mm, 9 mm, 19 mm, and 11 mm, respectively, against *F. solani* [126]. In another study, Perveen et al. (2022) reported hexadecanoic acid from diethyl ether extracts, which showed 73% growth inhibition of *F. oxysporum* and 50% growth inhibition of *F. Solani* [39].

The antifungal activity of unsaturated fatty acids, including monounsaturated fatty acids (MUFAs) and polyunsaturated fatty acids (PUFAs), is higher than that of saturated fatty acids. This is due to the presence of double bonds, which increase the fluidity of the cell membrane. All structural aspects of PUFAs, including shape, chain length, and the position and orientation of double bonds, contribute to their antifungal activity. Cis double bonds are particularly effective, as they induce greater membrane deformation compared to trans bonds due to their lower thermodynamic stability [34,133]. Isonitrile mirabile, a polyunsaturated fatty acid with a nitrogen atom as a halogenated compound, was isolated from the lipophilic extract of the cyanobacterium *Scytonema mirabile* and exhibited antifungal activity. Additionally, majusculoic acid, a PUFA containing bromine, was isolated from marine cyanobacteria and demonstrated antifungal properties [137]. Furthermore, Farghl and El-Sheekh (2019) reported PUFAs, which included tetradecanoic acid 12-methyl ester, 9,12-octadecadienoic acid methyl ester, and 9-octadecenoic acid methyl ester with antifungal activity against *F. oxysporum* and showed a 160 mm inhibition zone [124].

**Table 4:** Fatty acids from macro- and microalgae and cyanobacteria with antifungal activity against *Fusarium* spp.

Specie	Organism	Fatty Acids	Growth/Conditions	Solvent Extraction	Technique	Positive Drug Control	Result	References
*N. calcicola*	Cyanobacteria	9-octadecenoic acid Oleic acid 12,15-octadecadienoic acid methyl ester Palmitic acid	Batch 28 °C, 24 h light BG-11 medium	Methanol	GC/MS	NA	16 mm of inhibition zone mycelium of *F. oxysporum f.* sp. *lycopersici*	[138]
*N. carneum*	Cynobacteria	Palmitic acid 9,12-Octadecadienoic acid Alpha-Linoleic acid	Batch 30 °C, 12 h light BG-11 medium	Ethyl-acetate Ethanol	GC/MS	NA	3, 160 mm of inhibition zone mycelium of *F. oxysporum*	[124]
*Lyngnbya wollei*	Cyanobacteria	Gamma-Linoleic acid Palmitic acid 6,9,12,15-Octadecatetraenoicacid, methyl ester	Batch 28 °C 16 h light BG-11 medium	Methanol	GC/MS	NA	46% inhibition observed against *F. udum* and 40% against *F. oxysporum*	[107]
*Oscillatoria princeps*	Cyanobacteria	Alpha-Linoleic acid 9-Octadecenoic acid Hexadecanoic acid 9,15-Octadecadienoic acid 11-Octadecenoic acid	BG-11	Diethyl ether	GC/MS	Nystatin ${50,000\;\mu g\cdot mL}^{-1}$	Inhibition zone 14 mm *F. verticelloides* and 15 mm *F. proleferatum*	[140]
*C. myrica*. *P. boergesenii* *S. cinereum*	Macroalgae	Hexadecanoic acid, methyl ester 9-octadecenoic acid, methyl ester Tetradecanoic acid, methyl ester cis-11-Eicosenoic acid, methyl ester	NA	Methanol Acetone	GC/MS	Gentamycine and Ampicilline	Inhibition zone (mm) 15, 13, 14 by *C. myrica*, *S. cinereum* and *P. boergesenii* to *F. oxyporum*	[127]
*H. cuneiformis*	Macroalgae	Palmitic acid Myristic acid Stearic acid Oleic acid Palmitoleic acid	NA	Chloroform	GC/MS	Amphotericin B ${100\;\mu g\cdot mL}^{-1}$	6 µg/mL as MIC and 16 mm of inhibition zone of *F. oxysporum*	[139]
*C. sinuosa.* *P. pavonia*. *C. barbata*. *S.vulgare*	Macroalgae	Palmitic acid Myristic acid Palmitoleic acid Oleic acid Linoleic acid	NA	Methanol	GC/MS	Miconazole Flucanozole Itraconzaole ${100\;\mu g\cdot mL}^{-1}$	Inhibition zone diameters 19 mm, 9 mm, 19 mm, 11 mm by *C. sinuosa*, *P. pavonisa*, *C. barbata* and *S. vulgare*, respectively, to *F. solani*	[126]
*C. vulgaris*	Microalgae	Hexadecanoic acid Octadecenoic acid, methyl ester.	25 °C 24 h light BG-11 Medium	Diethyl ether	GC/MS	NA	Growth inhibition (%) 73 *F. oxysporum* 50 *F. solani*	[39]

GC/MS (gas chromatography/mass spectrometry); MIC (minimum inhibitory concentration); NA (not applicable).

## 5. Future Directions and Challenges

Current research has highlighted the potential of algal and cyanobacterial extracts as valuable sources of antifungal bioactive molecules [3]. Although general antimicrobial properties are well documented [141], there is limited research on their antifungal effects [137], particularly against phytopathogenic fungi like *Fusarium* spp.

In vitro methods for evaluating antifungal activity, including percentage inhibition and inhibition zones (mm), are well-established for plant extract [142]. However, studies on algae crude extracts often lack MIC (µg mL^−1^) determination, focusing mainly on inhibition percentages. This gap highlights the need for a more thorough evaluation of the antifungal potential of algae metabolites, particularly with phytopathogenic fungi.

Crude extracts are typically characterized by GC/MS, but this technique only detects volatile compounds and some fatty acids, often without derivatization [143]. Therefore, combining GC/MS with HPLC/MS is recommended to capture a broader range of metabolites, as these techniques reveal significantly different metabolite concentrations [144]. This dual approach would provide a more comprehensive view of the metabolites in the crude extract [145].

However, further studies are necessary to better understand the mechanisms by which these bioactive molecules interact with the *Fusarium* spp. cell wall [146]. The cell wall of *Fusarium* spp. consists of a glycoprotein-rich outer layer and a chitin/glucan matrix, which is critical for developing selective fungicides [147,148]. Metabolites such as phenolic acids, aromatic compounds, volatile compounds, and bioactive molecules like fatty acids have been shown to interact with the cell membrane and disrupt the cell wall [26,149,150]. Several hypotheses exist regarding how these metabolites affect the cell wall. First, bioactive molecules may form complexes with cell wall polymers, leading to conformational changes [114]. Second, antifungal biomolecules may penetrate the cell wall polymers and act directly on the ergosterol membrane [147]. Finally, these molecules may inhibit chitin synthase and β(1,3)-D-glucan synthase through competitive or non-competitive inhibition [151]. These mechanisms explain how bioactive molecules can interact with the cell wall, reach the cell membrane, and exert antifungal activity [148]. In line with these findings, environmental impact assessments of cyanobacterial and microalgal cultivation technologies, as well as large-scale seaweed harvesting, should be conducted to ensure the sustainability of these methods [141,152]. Scalability challenges in microalgal biomass use stem from the complexity and cost of harvesting and extracting bioactive metabolites. A study on producing 22,000 L of amphidinol-based fungicide in pilot-scale reactors found that adopting a biorefinery approach, which included co-products such as fatty acids and carotenoids, resulted in 34.61 tons of CO_2_ emissions over 15 years. This approach reduced the Global Warming Potential by 82–98% and demonstrated lower toxicity compared to commercial fungicides. Key improvements for commercial viability include enhancing bioreactors, reducing carbon footprint, using alternative media like wastewater, and optimizing extraction processes [153]. Moreover, the extraction and purification of high-value lipids, such as PUFAs, remain expensive and technologically demanding. While algal lipids exhibit useful functional properties, their performance can be inferior to low-molecular-weight surfactants in specific applications. Genetic modification of algae presents a promising strategy to enhance light utilization efficiency and increase lipid yield [154,155].

Nevertheless, although research on bioactive molecules with antifungal activity against *Fusarium* is still limited, most studies have focused on crude extracts using organic solvents, which range from non-polar to polar. These studies generally identify the chromatographic profile of bioactive compounds based on their polarity [3,156]. Researchers commonly use GC/MS as a chromatographic technique due to its ability to separate volatile bioactive molecules or those that can be derivatized, facilitating the identification of their structure. Fatty acid methyl esters, aromatic carboxylic acids, and volatile compounds can be detected using GC/MS, which is often preferred over HPLC for its robustness and ability to detect non-volatile compounds through derivatization [157,158]. However, both chromatography techniques are essential for a comprehensive analysis of all bioactive molecules in crude extracts [156]. Crude extract analysis is not conducted in isolation; compounds are fractionated and analyzed individually, which is crucial for identifying specific antifungal compounds [159]. This specificity is key to identifying and characterizing effective antifungal molecules [160].

Cyanobacteria such as *Nostoc* spp. and *Anabaena* spp. are known to synthesize cyclic peptides and lipopeptides with antifungal activity, but these molecules have mainly been studied in relation to human fungal pathogens like *Candida* spp. [161,162]. In contrast, phenolic acids are the most reported antifungal metabolites in eukaryotic microalgae and macroalgae [163]. Despite the known antifungal properties of these bioactive molecules in biological control, research on their effectiveness against phytopathogenic fungi is limited [35]. Future studies should first evaluate the antifungal activity of crude extracts against phytopathogenic fungi like *Fusarium* spp., followed by a determination of the profile of active compounds in the extract [164]. While this experimental approach is valid, future research should prioritize evaluating purified molecules such as fatty acids, phenolic acids, and cyclic peptides against phytopathogenic fungi, rather than relying solely on crude extract activity [161,162,165]. In this context, allelopathy in cyanobacteria and algae plays a crucial role by releasing metabolites with antibacterial and antifungal effects, which can be studied through co-cultivation to activate silent genes under stress [105,166]. Additionally, algae excrete volatile organic compounds with antifungal potential, and while their isolation is challenging, methods like counter-current chromatography are gaining interest for their high recovery and cost-effectiveness [112,167].

Existing chromatographic and spectroscopic profiles from studies on antifungal activity against *Fusarium* spp. suggest that fats and volatile compounds such as alkanes and hydrocarbons are the most important components with antifungal properties [104,137]. Since fatty acids are the most predominant bioactive compounds against *Fusarium* spp., they can be purified from oil extracts and further evaluated for their antifungal activity [168]. If their antifungal activity proves promising, additional studies will be needed to assess their potential in planta or in crop assays infected with *Fusarium* spp. [169]. Practical implementation of sustainable farming practices, advanced analytical tools, fractionation of extracts, investigation of well-known classes of antifungals, and a deeper understanding of the specificity of fatty acids are key directions for fully utilizing this natural resource in sustainable and effective applications [35,168,170]. Building on sustainable farming practices and advanced analytical techniques, we conclude that marine organisms offer bioactive compounds with potential as biofungicides. Understanding their pharmacokinetics, including absorption, distribution, metabolism, and excretion, is essential for optimizing their application in crops for human consumption [171]. Studies on *Schizochytrium* sp. oil demonstrated over 90% cell viability in Caco-2 cells after 48 h of exposure to 200 µg/mL of DHA, and acute toxicity in vivo tests classified it as category 5 under OECD 423 guidelines, with the highest safety level at 2000 mg/kg [172]. Furthermore, nanoencapsulation of fucoxanthin from *Phaeodactylum tricornutum* significantly improved metabolite absorption, although challenges persist, such as the low oral bioavailability of compounds like astaxanthin (90.1% lower compared to intravenous administration) [173]. These findings suggest algal metabolites may improve the safety of marine-derived biofungicides in agriculture by ensuring low oral bioavailability. However, further studies on their pharmacology in animals are needed to ensure safe and effective use. In parallel, the integration of Artificial Intelligence (AI) in discovering antifungal metabolites, such as cyclic peptides and lipopeptides, is a growing trend [174]. AI techniques like Generative Adversarial Networks, Variational Autoencoders, Recurrent Neural Networks, and Graph Neural Networks can encode peptide prototypes and generate analogs through latent space sampling [175,176]. Additionally, AI-based databases such as GNPS, NPAtlas, and DNP help identify fragmentation patterns and unique compounds, streamlining the antifungal screening process by dereplicating known compounds and prioritizing novel antifungal metabolites [166].

## 6. Conclusions

Macroalgae, microalgae, and cyanobacteria are emerging as sustainable sources of natural antifungal agents, with crude polar extracts demonstrating notable antifungal activity against *Fusarium* spp. This is evidenced by metrics such as percentage inhibition, mycelial inhibition zones, and growth rates. Although the fungicidal effects have not been thoroughly investigated, these results present a valuable opportunity to fractionate the extracts, identify minimum inhibitory concentrations (MICs), and perform additional assays to gain a deeper understanding of the mechanisms involved. Investigating variations in ergosterol and chitosan levels could provide insights into whether these bioactive compounds influence fungal membranes and cell walls. Gas chromatography analysis has pinpointed fatty acids as significant bioactive metabolites, indicating their potential for purification and separate evaluation. Furthermore, phenolic compounds and other metabolites deserve more attention. The ability of algae and cyanobacteria to thrive in wastewater and saline conditions and to sequester CO_2_ enhances their appeal for biocontrol applications, while lower cultivation costs are required as compared to terrestrial plants. This review serves as the first extensive compilation of research on the antifungal properties of algae and cyanobacteria extracts, particularly against *Fusarium* spp., establishing a foundation for future studies that could transform agricultural practices by providing eco-friendly and cost-effective solutions for tackling fungal pathogens.

## Figures and Tables

**Table 2:** Overview and comparison of commercial biofungicides for sustainable agricultural practices.

Commercial Product	Active Ingredients	Category	Claimed Mode of Action	WEB Page
Serenade Garden Disease Control	*B. subtilis* strain QST 713	BCAs	Colonization of plant roots and production of antifungal compounds	1
Companion^®^ Biofungicide	*Bacillus amyloliquefaciens* strain D747	BCAs	Production of antifungal metabolites	2
Trilogy^®^ Biofungicide	*B. subtilis* strain QST 713 and *Pseudomonas fluorescens* strain Pf-L13	BCAs	Colonization of plant roots and production of antifungal compounds	3
RootShield^®^ 10G Biofungicide	*Trichoderma harzianum* strain 1295-22	BCAs	Parasitism of fungal pathogens and production of antifungal metabolites	4
Actinovate^®^ SP	*Streptomyces lydicus* strain WRLDac951	BCAs	Production of multiple antifungal compounds	5
Prothio T ^®^	Prothioconazole	Chemical Fungicides	Inhibition of ergosterol biosynthesis in fungal cell membranes	6
Headline^®^	Strobilurin (multiple formulations)	Chemical Fungicides	Inhibition of mitochondrial electron transport in fungal cells	7
Switch^®^	Fludioxonil	Chemical Fungicides	Disruption of fungal cell wall biosynthesis	8
Tebuconazole 25 EW	Tebuconazol	Chemical Fungicides	Inhibition of ergosterol biosynthesis in fungal cell membranes	9
Phyton-27^®^	*Laminaria digitata* extract	BCAs	Disruption of fungal cell membranes and suppression of spore germination	10
Kocide^®^ 2000	Copper oxychloride	Broad spectrum, including fungal spores and mycelia	Disruption of fungal cell membranes and enzyme inhibition	11

BCA (Biological Control Agents): (1) https://www.planetnatural.com/product/serenade-garden-disease-control/ (accessed on 22 March 2024); (2) https://link.springer.com/article/10.1007/s13744-021-00939-2 (accessed on 22 March 2024); (3) https://trinitybiochem.com/ (accessed on 25 March 2024); (4) https://www.biocontrols.com/secure/shop/listcats.aspx (accessed on 25 March 2024); (5) https://www.novozymes.com/en/products/bioag/biocontrol/actinovate-sp-us (accessed on 25 March 2024); (6) https://www.genfarm.com.au/crop-protection/fungicides/genfarm-prothio-t-fungicide (accessed on 29 March 2024); (7) https://agriculture.basf.com/mx/es/proteccion-de-cultivos-y-semillas/productos/headline.html (accessed on 29 March 2024); (8) https://www.syngenta.com.mx/product/crop-protection/fungicida/switchr-625-wg (accessed on 29 March 2024); (9) https://www.sag.gob.cl/sites/default/files/resol._renov._tebuconazole_25_ew_solchem_spa-etiqueta.pdf (accessed on 29 March 2024); (10) https://www.imex.mx/producto/phyton-27/ (accessed on 29 March 2024); (11) https://www.corteva.in/products-and-solutions/crop-protection/kocide-2000.html (accessed on 29 March 2024).

**Table 3:** Antifungal metabolites extracted from biomass of macro-, micro-, and cyanobacteria.

Species	Organism	Compound	Structure /Type	Solvent	Method	Positive Drug Control	Results	Reference
*Spirulina* sp.	Cyanobacteria	Chlorogenic acid Protocatechuic acid Gallic acids	Phenolics	Water	LC/ESI/MS/MS	Synthetic acid phenolic standard	Mycelial growth rate was lowest as 0.21 cm·day^−1^ *F. asiaticum*, *F. graminearum* and *F. meridionale*	[122]
*Spirulina* sp.	Cyanobacteria	Gallic acid Caffeic acid Salicylic acid	Phenolics mixture	Methanol	HPLC-UV	NA	Mycelial growth rates ranged from 0.01 cm to 0.45 cm·day^−1^ for *F. meridionale* and *F. graminearum*	[123]
*N. calcicola*	Cyanobacteria	Phthalic acid Xylene	Aromatic carboxylic acids and aromatics	Methanol	GC/MS	NA	16 mm of inhibition zone mycelium of *F. oxysporum* and *F. lycopersici*	[38]
*N. carneum*	Cyanobacteria	Phytol	Terpenoids	Ethyl acetate	GC/MS	Chloramphenicol	3, 160 mm of inhibition zone mycelium of *F. oxysporum*	[124]
*Spirulina platensis*	Cyanobacteria	2-Hexyl-1-nitrocyclohexane Bromoacetic acid	Nitroalkane bromine compounds	Methanol:acetone:diethyl eter (5:2:1)	GC/MS	NA	Strong antifungal activity for *F. oxysporum* at 50,000 µg/mL concentration of extract.	[125]
*S.dentifolium* *G. compressa* *U. lactuca*	Macroalgae	Phloroglucinol Gallic acid Vanillic acid	Phenolics	Methanol	HPLC	NA	Diameter of mycelial growth (mm) 41, 49, 45 at 20 µg/mL extract respectively to against *F. oxysporim* f sp. *lycopersici*	[30]
*Colpomenia sinuosa*. *Padina pavonia*. *Cystoseira barbata*. *Sargassum vulgare*.	Macroalgae	Hexadecanol Heptacosane diisooctyl phthalate Butyl octyl phthalate	Aromatic carboxylic acids and Volatiles	Methanol	GC/MS	Miconazole Flucanozole Itraconzaole ${100\;\mu g\cdot mL}^{-1}$	Inhibition zone diameters 19 mm, 9 mm, 19 mm, and 11 mm, respectively, against *F. solani*	[126]
*Dunaliella* sp. *Chlorella sorokiniana*	Microalgae	Gallic acid Quercetine Equivalents	Phenolics and alkaloids	Methanol	UV/VIS	Rhizolex-T ${20\;\mu g\cdot mL}^{-1}$	Inhibition (%) 82, 40 by *Dunaliella* sp and *C. sorokiniana* to *F. solani*	[127]
*Navicula arenaria*	Microalage	di-n-octyl phthalate Beta-Sitosterol	Aromatic carboxylic acids, sterols	Hexane	GC/MS	Miconazole And Nystatin	Mycelium growth inhibition 22% at 6000 µg/mL of hexane extract to *F. oxysporum*	[128]
*Nannochlorosis* sp.	Microalage	Chlorogenic acid Gallic acid Protocatechuic	Phenolics acids	Methanol	HPLC/MS	Synthetic acid phenolic standard	Mycelium Growth rate lowest < 0.32 cm·day^−1^ for *F. asiaticum*, *F. graminearum*, *F. meridionale.*	[122]

ESI (electrospray ionization), GC/MS (gas chromatography/mass spectrometry), HPLC (high-performance liquid chromatography), MS (mass spectrometry), LC (liquid chromatography), UV (ultraviolet), and VIS (visible), NA (not applicable).
